# Characteristics and Phylogenetic Analysis of the Complete Chloroplast Genome of *Abelmoschus esculentus*

**DOI:** 10.3390/ijms27010118

**Published:** 2025-12-22

**Authors:** Junyuan Dong, Guanghui Du, Qingqing Ji, Xingcai An, Ziyi Zhu, Shenyue Tang, Xiahong Luo, Changli Chen, Tingting Liu, Lina Zou, Shaocui Li, Jiquan Chen, Xia An

**Affiliations:** 1 School of Agriculture, Yunnan University, Kunming 650500, Chinadgh2012@ynu.edu.cn (G.D.);; 2 Zhejiang Xiaoshan Institute of Cotton & Bast Fiber Crops, Zhejiang Institute of Landscape Plants and Flowers, Zhejiang Academy of Agricultural Sciences, Hangzhou 311251, China; 3 College of Environment and Resources, College of Carbon Neutrality, Zhejiang A&F University, Hangzhou 311300, China; 4 Institute of Bast Fiber Crops, Chinese Academy of Agricultural Sciences/Key Laboratory of Bast Fiber Biology and Processing, Ministry of Agriculture and Rural Affairs, Changsha 410221, China

**Keywords:** *Abelmoschus esculentus*, chloroplast genome, taxonomy, phylogenetic analysis

## Abstract

*Okra* (*Abelmoschus esculentus* L. Moench) is an annual herbaceous plant belonging to the Malvaceae family. Its medicinal properties and edible value have attracted widespread scientific attention, yet its systematic taxonomy, evolution, and photosynthetic mechanisms warrant further investigation. Chloroplasts, specialized semi-autonomous organelles within green plants, possess their own genetic material and serve as an excellent source of genetic information. This study employed Illumina high-throughput sequencing technology to sequence the complete chloroplast genome of the cultivar ‘Gan Kui No. 1’. The complete chloroplast genome was determined to be 163,121 bp in length, with A, C, G, T, and GC nucleotides accounting for 31.24%, 18.71%, 18.02%, 32.02%, and 36.74% of the total, respectively. It exhibits a classic tetrad structure, comprising one large single-copy region (88,071 bp), one small single-copy region (19,032 bp), and one pair of inverted repeat regions (28,009 bp). The entire chloroplast genome contains 132 annotated genes, including 37 tRNA genes, 8 rRNA genes, 87 mRNA genes, and 0 pseudogenes. A phylogenetic tree constructed using 20 species, including *Abelmoschus esculentus*, revealed a clear phylogenetic relationship between the genus *Hibiscus* and *Abelmoschus esculentus*. The complete gene sequences have been uploaded to the NCBI database (accession number PX590535). This study provides insights into understanding the evolutionary relationships of *Abelmoschus esculentus* and refining its taxonomy, laying a theoretical foundation for subsequent research on the *Abelmoschus esculentus* chloroplast genome.

## 1. Introduction

Okra (*Abelmoschus esculentus* L. Moench) is an annual herbaceous plant belonging to the Malvaceae family. It is now widely distributed throughout tropical, subtropical, and warm temperate regions worldwide [1]. *Abelmoschus esculentus* has multiple uses for its tender leaves, flower buds, flowers, pods, stems, and seeds [2] due to its richness in bioactive compounds such as flavonoids, polyphenols, polysaccharides, and amino acids. It possesses multiple bioactive substances [3]. In addition to direct consumption as a nutritious vegetable, *Abelmoschus esculentus* can also serve as a food additive by extracting functional components, such as *Abelmoschus esculentus* gum, exhibiting excellent emulsifying stability [4].

Chloroplasts are metabolically active, semi-autonomous organelles found in plants, algae, and cyanobacteria [5]. Although photosynthesis is commonly regarded as the primary function of plastids, they play crucial roles in plant genetic evolution and the synthesis of amino acids, vitamins, and numerous metabolites [6,7]. The chloroplast genome holds significant importance in the study of biological evolution and taxonomy [7,8]. Historically, there has been debate regarding the origin and taxonomic classification of *Abelmoschus esculentus*. Discussions have centered on whether the genus *Abelmoschus* should be incorporated into the genus *Hibiscus* or recognized as a distinct genus [9]. The advent of modern genetic analysis technologies, such as Illumina high-throughput sequencing, has accelerated rapid progress in the fields of chloroplast genetics and genomics. Increasing evidence supports the taxonomic classification of *Abelmoschus esculentus* [10]. In 2020, Jie Li and colleagues conducted a systematic study of the chloroplast genome of *Abelmoschus esculentus*, providing crucial insights for species identification and the exploration of *Abelmoschus esculentus*’s origin and evolution [11]. Notably, Liu et al. (2023) recently presented and compared the chloroplast genomes of three okra varieties, confirming structural conservation and a close phylogenetic affinity with *Talipariti hamabo* [12]. The high-quality chromosome-level nuclear genome of okra provides groundbreaking insights into studying genome duplication events and the genetic basis of nutrient metabolism [13].

However, these studies remain largely descriptive, focusing on genome assembly, annotation, and preliminary phylogenetic placement. They lack in-depth evolutionary analyses that could reveal the forces shaping the chloroplast genome, such as the identification of genes under natural selection, a comprehensive assessment of sequence variation and codon usage bias, and analysis of structural changes like inverted repeat (IR) boundary shifts in a broader phylogenetic context.

To bridge these gaps and move beyond a simple descriptive account, this study employs high-throughput sequencing and comprehensive bioinformatics analyses on the cultivar ‘Gan Kui No. 1’. Our objectives are not only to report the genome sequence but to perform an in-depth evolutionary investigation, including codon usage bias analysis to infer translational selection; identification of simple sequence repeats (SSRs) and dispersed repeats to assess mutation dynamics; Ka/Ks analysis to pinpoint genes under positive or purifying selection, potentially linked to environmental adaptation; nucleotide diversity (Pi) analysis to locate hypervariable regions useful for marker development; detailed IR boundary analysis to understand structural evolution; and a robust phylogenetic reconstruction using a broad set of Malvaceae species to clarify the systematic position of Abelmoschus. This study will utilize the *Abelmoschus esculentus* cultivar ‘Gan Kui No. 1’ to conduct whole-genome sequencing, assembly, and annotation of the *Abelmoschus esculentus* chloroplast genome through high-throughput sequencing technology and bioinformatics methods. This study selected the okra cultivar ‘Gan Kui No. 1’ as experimental material based on the following considerations: First, ‘Gan Kui No. 1’ is a widely cultivated, high-yielding, high-quality mainstay variety. Analyzing its chloroplast genome holds direct practical value for guiding molecular breeding in this cultivar. Second, this material is a germplasm resource extensively studied by our research group, possessing comprehensive agronomic trait data, which facilitates subsequent association analysis between genomic features and phenotypic traits. The study aims to provide fundamental theoretical support for the phylogenetic analysis of the genus Hibiscus, the development of new cultivars, and the utilization of novel germplasm resources.

## 2. Results

### 2.1. Basic Characteristics of the Chloroplast Genome of Abelmoschus esculentus

**Figure 1:**
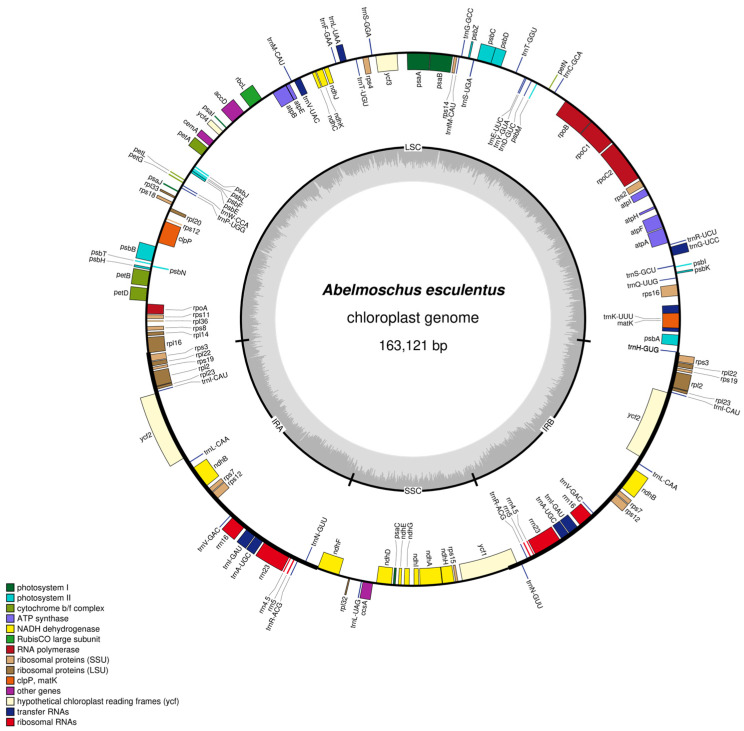
Circular map of the chloroplast genome of *Abelmoschus esculentus* cv. ‘Gan Kui No. 1’. The map was generated using OGDRAW (v1.3.1). Genes on the outside of the circle are transcribed clockwise, while inner genes are transcribed counterclockwise. The inner gray histogram illustrates the GC content, highlighting the elevated GC content in the inverted repeat (IR) regions compared to the single-copy regions.

The chloroplast genome of *Abelmoschus esculentus* exhibits a common tetrameric structure with a total length of 163,121 bp. The nucleotide composition shows A, C, G, T, and GC account for 31.24%, 18.71%, 18.02%, 32.02%, and 36.74% of the total, respectively. Notably, the proportions of C and G are significantly lower compared to the others. The genome comprises four segments: large single-copy regions (LSC) and small single-copy regions (SSC), inverted repeat sequence a (IRa) and inverted repeat sequence b (IRb), with respective lengths of 88,071 bp, 19,032 bp, 28,009 bp, and 28,009 bp (Figure 1, Table 1). Within the large single-copy region (LSC), GC content was highest at 34.55%, with a total size of 30,425 bp. In the small single-copy region (SSC), T had the highest proportion at 34.64%, with a total size of 6593 bp. In the inverted repeat sequence a (IRa), GC had the highest proportion at 41.97%, with a total size of 11,754 bp.

### 2.2. Functional Annotation of Chloroplast Genes

Genes are functionally categorized into four major groups: photosynthesis, self-replication, other genes, and genes of unknown function. A total of 132 genes were identified, including 37 tRNA genes, 8 rRNA genes, 87 mRNA genes, and 0 pseudogenes.

Among the genes involved in photosynthesis, photosystem I (Subunits of photosystem I) includes genes such as *psaA*, *psaB*, and *psaC*, while photosystem II (Subunits of photosystem II) encompasses numerous genes, including *psbA*, *psbB*, and *psbC*, demonstrating the well-developed molecular basis for photosynthesis in *Abelmoschus esculentus*. Additionally, numerous other pathways are involved, such as photosynthetic electron transport, ATP synthesis, and carbon fixation. These include NADH subunits (*ndhA*, *ndhB*, etc.), cytochrome subunits (*petA*, *petB*, etc.), ATP synthase subunits (*atpA*, *atpB*, etc.), and the large subunit of ribulose-1,5-bisphosphate carboxylase (*rbcL*). Together, these components ensure the efficient execution of photosynthesis (Table 2).

Numerous genes participate in self-replication, including *rpl14* and *rps11* associated with large and small subunits, *rpoA* and *rpoB* associated with RNA polymerase subunits, *rrn16* and *rrn23* associated with ribosomal RNA, and trnA-UGC and trnC-GCA associated with transfer RNA. These form the molecular basis for chloroplast replication, transcription, and translation.

Simultaneously, numerous genes involved in other functions were detected, and some functions of genes within the chloroplast genome remain unidentified, leaving room for further functional studies.

### 2.3. Codon Preference Analysis

A systematic analysis of codon usage characteristics in the chloroplast genome of *Abelmoschus esculentus* revealed a total of 22,797 codons. Codon preference analysis indicated a strong preference for the stop codon UAA (RSCU = 1.6539), with lower usage of UAG (0.6537) and UGA (0.6924). Statistics show that 19 amino acids are formed. Regarding codons involved in amino acid coding: Ala (Alanine) prefers GCU (RSCU = 1.7828); Cys (Cysteine) prefers UGU (1.504); Asp (Aspartic Acid) prefers GAU (1.5938); glutamic acid (Glu) favors GAA (1.508); lysine (Lys) favors AAA (1.5088); arginine (Arg) favors AGA (1.7616) and CGA (1.3998); serine (Ser) favors UCU (1.7136) and UCA (1.263); Tyrosine (Tyr) favors UAU (1.5982) Lysine (Lys) favors AAA (1.5088); Leucine (Leu) favors CUU (1.2282), UUA (1.9692), UUG (1.2252) (Table 3). The methionine (Met) codons AUA, AUC, and AUU all have a score of 0, with only AUG (RSCU = 7) being a valid codon. Overall, there is a preference for codons ending in A or U, reflecting the species’ bias in the nucleotide composition of its codons. Among the 19 amino acids encoded, the overall codon usage exhibited a marked bias towards codons ending with A or U (Table 3). For instance, Ala (Alanine) prefers GCU (RSCU = 1.7828); Phe (Phenylalanine) prefers UUU (1.3422); and Lys (Lysine) prefers AAA (1.5088).

**Figure 2:**
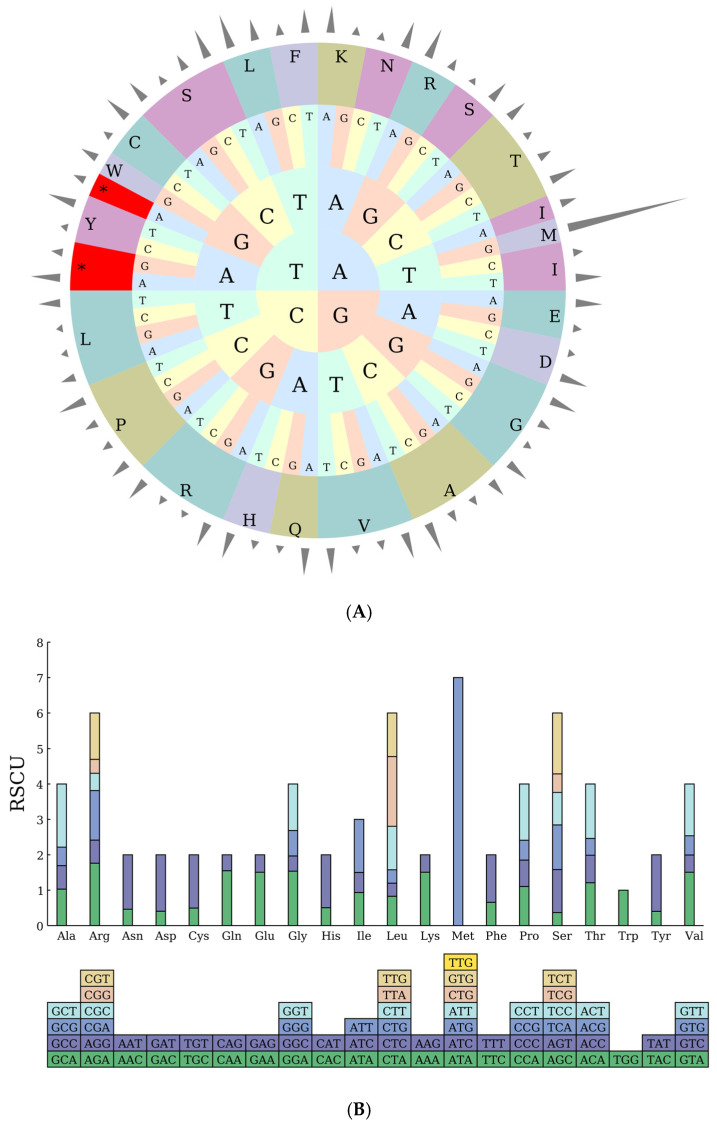
(**A**) RSCU Pie Chart. Note: The outermost cylinder represents the RSCU value, the middle layer consists of amino acids, and the innermost three layers represent codons. Different colors denote different amino acids (abbreviations are labeled on the outer ring, e.g., L for leucine, F for phenylalanine, etc.); the inner letters (A, T, C, G) indicate nucleotides; asterisks (*) mark codons with significant characteristics. (**B**) RSCU Histogram. Note: The squares below represent all codons encoding each amino acid, while the height of the columns above represents the total sum of RSCU values for all codons.

The RSCU pie chart (Figure 2A) and bar chart (Figure 2B) generated from the above data visually depict the distribution patterns of amino acid-specific codons, aiding in the interpretation of codon usage preferences within the *Abelmoschus esculentus* chloroplast genome.

### 2.4. Repeat Sequence Analysis

**Figure 3:**
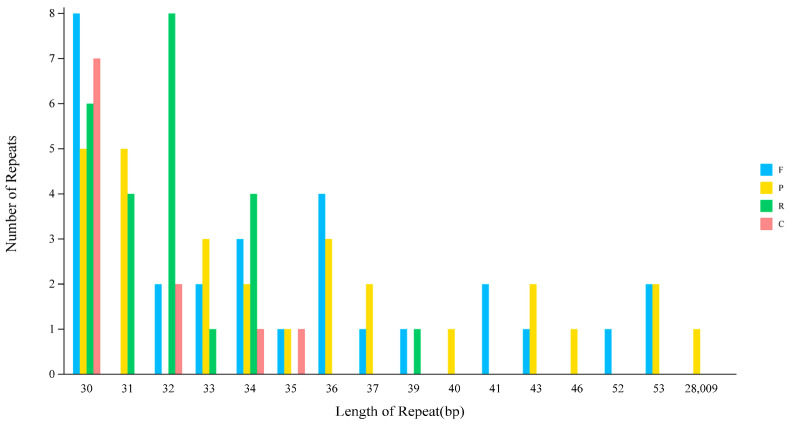
Analysis of scattered sequence repeats in the chloroplast genome of *Abelmoschus esculentus* L. Note: The horizontal axis represents the length of scattered repetitive sequences, while the vertical axis represents the number of scattered repetitive sequences. F denotes forward repeats, P denotes palindromic repeats, R denotes reverse repeats, and C denotes complementary repeats.

Through repetitive sequence analysis, lengths ranged from 30 bp to 28,009 bp, with the predominant length cluster being approximately 30–37 bp. Long repetitive sequences were rare. The most abundant repeat length was 30 bp (26 sequences), followed by 32 bp (12 sequences) and 34 bp (10 sequences). Larger sequences (e.g., 52 bp, 53 bp, 28,009 bp) were extremely rare, with only 1–4 sequences each. Forward repeats (F) and palindromic repeats (P) were the most abundant, each with 28 occurrences. Reverse repeats (R) numbered 24, while complementary repeats (C) were the least frequent, with only 11 instances. A bar chart provides a more intuitive representation of the quantitative differences among repeats of varying lengths and types. The quantities of forward repeats (F), palindromic repeats (P), reverse repeats (R), and complementary repeats (C) all remained at relatively high levels (Figure 3).

Analysis identified 91 dispersed repeats in the chloroplast genome of Abelmoschus esculentus, with lengths ranging from 30 bp to 28,009 bp (Table 4). Short repeats (30–37 bp) predominated, constituting 72.5% of the total, with 30-bp repeats being the most abundant (26 sequences). In terms of repeat types, forward (F) and palindromic (P) repeats were the most common (28 each), followed by reverse (R, 24) and complementary (C, 11) repeats (Figure 3).

### 2.5. Simple Sequence Repeat (SSR) Analysis

Analysis revealed the presence of 344 SSRs in the chloroplast genome of *Abelmoschus esculentus*, comprising 236 large single-copy regions (LSC), 44 small single-copy regions (SSC), and 32 each in the two inverted repeat sequences (IRa and IRb).

Among the five base-pair repeat types, single-base repeats were the most frequent, occurring 207 times. This was followed by triplet repeats (74 occurrences). Other base-pair repeats included: diplet repeats (21 occurrences), quadruplet repeats (7 occurrences), quintuplet repeats (2 occurrences), and hexuplet repeats (1 occurrence).

**Figure 4:**
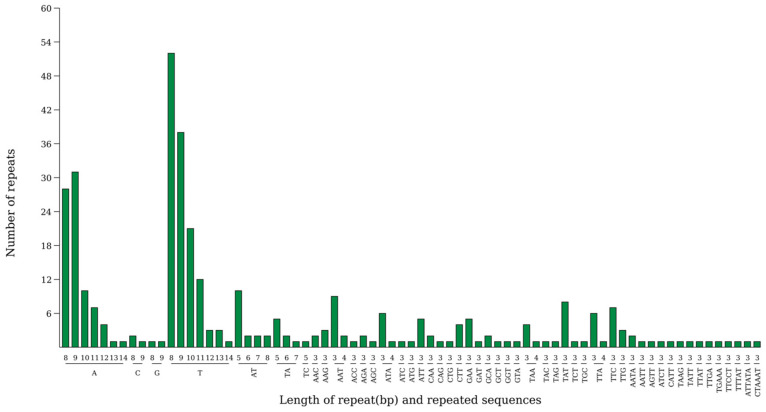
Analysis of simple sequence repeats in the chloroplast genome of *Abelmoschus esculentus* L. Note: The horizontal axis represents SSR repeat units, and the vertical axis represents the number of repeat units.

The vast majority of cpSSRs are short sequences, consistent with the structural characteristics of the chloroplast genome. SSRs ranging from 10 to 20 bp in length are the most abundant. A sharp decrease in SSR frequency is observed with increasing length. Region A (shortest) contains the highest number of SSRs, followed by Region B, while Regions C and D (longer) exhibit very few SSRs (Figure 4).

A total of 344 SSRs (cpSSRs) were identified in the chloroplast genome of Abelmoschus esculentus, with an uneven distribution: 236 in the LSC region, 44 in the SSC region, and 32 in each IR region (IRa and IRb). Mononucleotide repeats were the most frequent (207 occurrences), followed by triplet repeats (74). The abundance of SSRs decreased sharply with increasing repeat unit length and motif size (Figure 4).

### 2.6. Ka/Ks Analysis

Ka reflects the mutation frequency causing amino acid changes, while Ks reflects the mutation frequency without amino acid changes; Ka/Ks > 1 indicates positive selection, Ka/Ks < 1 indicates purifying selection, and Ka/Ks = 1 indicates neutral evolution.

**Figure 5:**
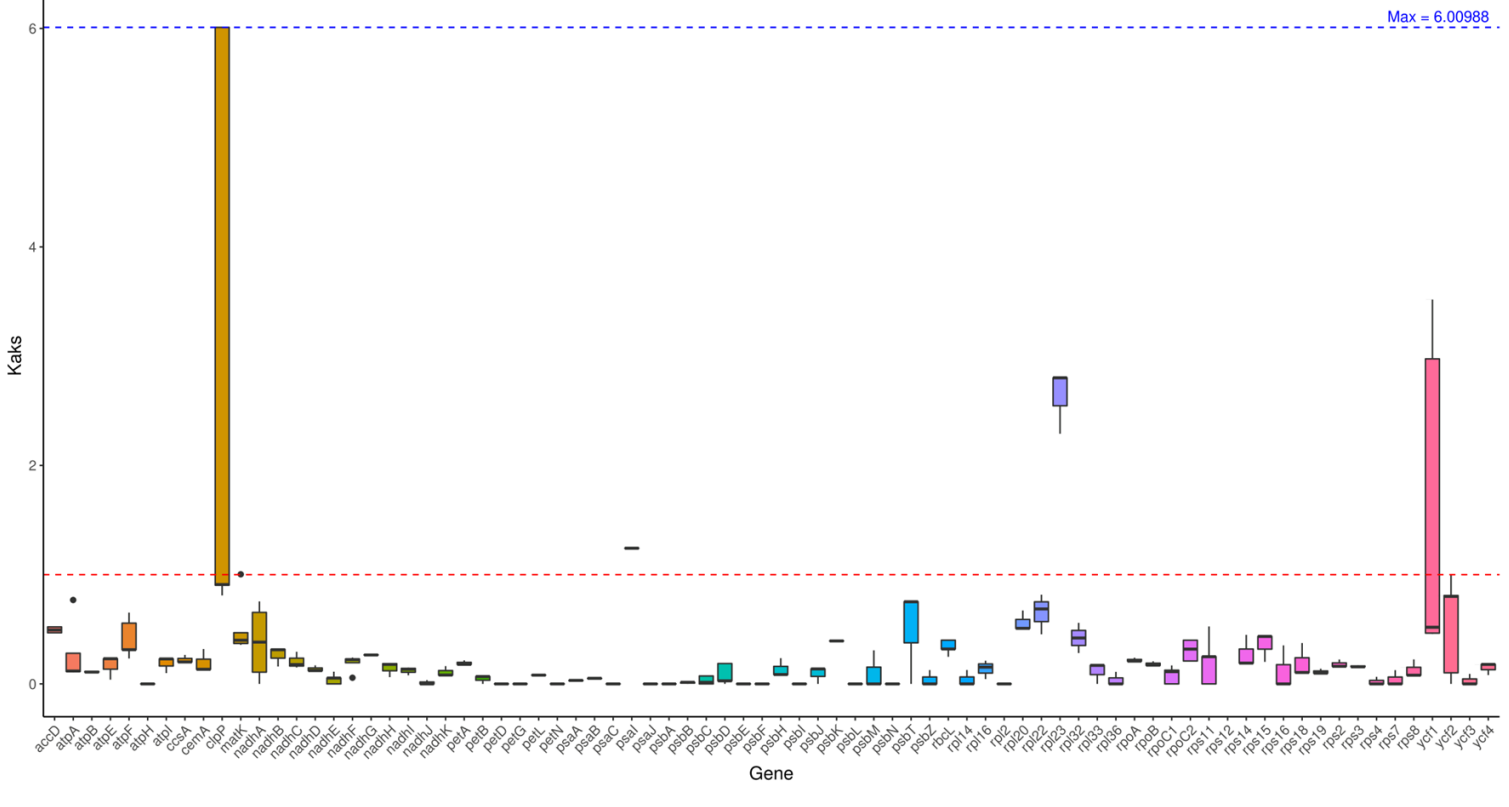
Ka/Ks analysis. Note: The horizontal axis represents gene names, while the vertical axis denotes Ka/Ks ratios. In the box plot, the upper and lower endpoints of the vertical lines above and below the rectangle indicate the upper and lower bounds of the data, respectively. The thick line within the rectangle represents the median, while the upper and lower edges of the rectangle denote the upper and lower quartiles. Data points extending beyond the upper and lower bounds of the rectangle are considered outliers.

Genes such as *atpF* and *rpoC2* exhibit Ka/Ks values greater than 1, indicating they have undergone positive selection during evolution. Most genes show Ka/Ks values significantly below 1, suggesting they are subject to strong purifying selection. Genes under positive selection may contribute to *Abelmoschus esculentus* adaptive evolution to environmental conditions, while those under purifying selection likely perform functions critical to the plant’s survival and reproduction (Figure 5).

The ratios of non-synonymous (Ka) to synonymous (Ks) substitution rates were calculated to assess selective pressures on protein-coding genes. Genes such as atpF (ATP synthase subunit) and rpoC2 (RNA polymerase subunit) exhibited Ka/Ks values greater than 1 (Figure 5), indicating they have likely undergone positive selection during evolution. In contrast, the vast majority of genes showed Ka/Ks values significantly below 1, suggesting strong purifying selection to maintain their essential functions.

### 2.7. Nucleic Acid Diversity Pi Analysis

The Pi value reflects the degree of genetic variation in genomic sequences: a higher Pi value indicates greater nucleotide diversity and stronger genetic variation in that region. The chloroplast genome comprises three parts—the large single-copy region (LSC), small single-copy region (SSC), and inverted repeat region (IR)—with a total of 111 gene regions detected. The Pi distributions of the three regions show distinct differentiation. The LSC region exhibits multiple Pi peaks and overall abundant variation, indicating high genetic diversity. The SSC region exhibits the highest Pi peak (approaching 0.032), indicating substantial nucleotide variation and active evolutionary activity. The IR region shows extremely low Pi values overall, reflecting its highly conserved structure as an inverted repeat sequence with stringent constraints on variation, demonstrating the structural stability of the chloroplast genome.

**Figure 6:**
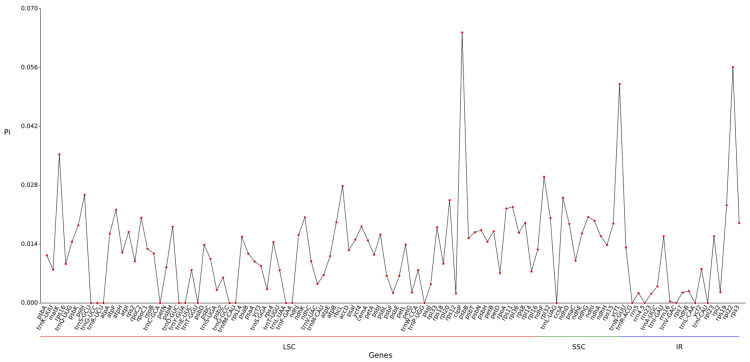
Line chart of gene Pi value.

Nucleotide diversity (Pi) analysis across the chloroplast genome revealed distinct patterns among the LSC, SSC, and IR regions (Figure 6). The SSC region exhibited the highest Pi peak (approaching 0.032), indicating it is a hypervariable region with substantial nucleotide variation. The LSC region also showed multiple Pi peaks, reflecting considerable genetic diversity. In contrast, the IR region displayed uniformly low Pi values, underscoring its highly conserved nature due to copy correction between the two inverted repeats.

Pi characteristics of different sequences exhibit distinct patterns: tRNA-related sequences such as *trnH* and *trnK* show higher Pi values in tRNA gene regions, indicating these areas possess abundant variation and may have contributed to genomic evolutionary differentiation. For protein-coding genes, Pi values vary significantly across functional groups. Genes involved in photosystem (*psa*, *psb* families) and ribosomal proteins (*rpl*, *rps* families) exhibit mixed patterns: some show high Pi values (high variation) while others display low Pi values (constrained by purification selection), reflecting divergent evolutionary pressures on different functional genes (Figure 6).

### 2.8. Boundary Analysis

**Figure 7:**
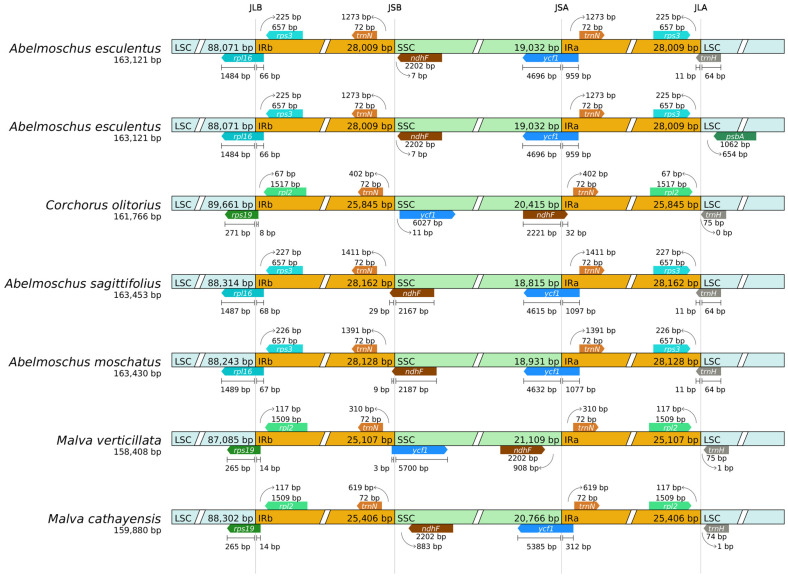
IR/SC boundary analysis.

The chloroplast genome adopts a circular structure, with four boundaries between IR and LSC/SSC: LSC-IRb, IRb-SSC, SSC-IRa, and IRa-LSC. The LSC-IRb boundary contains genes such as rpl16 and rps3, exhibiting stable gene-boundary spacing/overlap lengths—e.g., rpl16 is separated from IRb by 66 bp. The ndhF gene at the IRb-SSC boundary partially extends into IRb (spanning 7 bp across the boundary). At the SSC-IRa boundary, the ycf1 gene spans the boundary. At the IRa-LSC boundary, the trnH gene is separated from the boundary by 64 bp (Figure 7).

The length variation in chloroplast genomes is primarily driven by expansion/contraction of the intergenic regions (IRs). Within the genus Abelmoschus, species such as *Abelmoschus sagittifolius* (total length 163,453 bp) and *Abelmoschus moschatus* (total length 163,430 bp) have an IR region length similar to that of Abelmoschus esculentus. The overall genome length and distribution of boundary genes also show high similarity, reflecting the close phylogenetic relationships among species within the genus Abelmoschus and the low degree of structural differentiation in the IR region. For species from different genera, *Corchorus olitorius* (total length 161,766 bp) exhibits an IR region of only approximately 25,845 bp, while the SSC region spans 20,415 bp, demonstrating evolutionary characteristics of IR region contraction and SSC region expansion. *Malva verticillata* (158,408 bp) has an IR region of only about 25,107 bp, with a significantly shorter total length resulting from substantial IR region contraction (Figure 7).

Expansion/contraction of the IR region leads to changes in the distribution of boundary genes, which is one of the core characteristics of species evolution. These differences directly reflect evolutionary relationships among species. Species within the genus Abelmoschus exhibit greater similarity in IR region structure and boundary gene distribution, indicating closer phylogenetic relationships. In contrast, species from the genera Malva and Corchorus show significant differences in IR region length and boundary gene distribution, reflecting more distant evolutionary relationships. The expansion/contraction of the IR region serves as a primary driver of chloroplast genome evolution. These structural differences provide crucial genomic evidence for phylogenetic analysis and species classification identification.

Comparative analysis of the junctions between the inverted repeat (IR) and single-copy (LSC/SSC) regions among five Malvaceae species revealed dynamic expansion and contraction of the IR region (Figure 7). The IR region in *Abelmoschus esculentus* (28,009 bp) is significantly longer than that in *Gossypium hirsutum* (25,845 bp) and *Malva verticillata* (25,107 bp), due to the extension of genes like *ndhF* and *ycf1* across the IR-SSC boundaries. Congeneric species (*A. esculentus*, *A. moschatus*, *A. sagittifolius*) exhibited highly similar IR/SC boundary structures.

### 2.9. Phylogenetic Analysis

By constructing a phylogenetic tree using conserved coding sequences (CDS), this study investigated the evolutionary relationships among *Abelmoschus esculentus*, its conspecifics, other species within the same family, and outgroup species. Results indicate that okra (*Abelmoschus esculentus*) clusters with its congeneric species *Abelmoschus sagittifolius* and *Abelmoschus moschatus* within a single clade, supported by a 100% bootstrap value, confirming the extremely close phylogenetic relationship among species within the genus Abelmoschus. Similarly, *Hibiscus syriacus* (Hibiscus genus) clustered with *Abelmoschus esculentus* genus species with 100% support, indicating that the *Abelmoschus esculentus* and Hibiscus genera represent the most closely related groups within the Malvaceae family.

Within the Malvaceae family, the groups most closely related to the *Abelmoschus esculentus* and hibiscus lineages are, in order, the cotton genus, such as Gossypium hirsutum and Gossypium barbadense; Malva, such as Malva cathayensis and Malva verticillata. Each branch exhibits self-support rates ≥ 95%, reflecting the evolutionary relationships among Malvaceae taxa.

**Figure 8:**
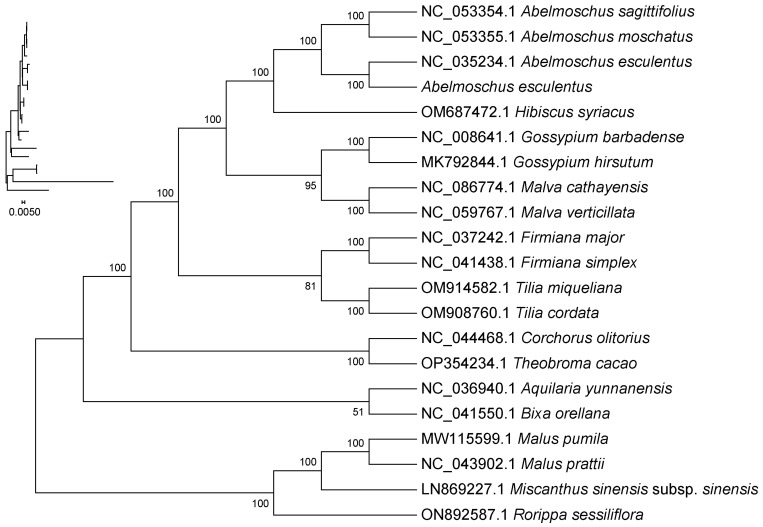
Phylogenetic tree constructed based on chloroplast genome sequences. Four species (*Malus pumila*, *Malus prattii*, *Miscanthus sinensis*, and *Rorippa sessiliflora*) were used as the outgroup. Note: (1) Sequence names correspond to species Latin names. (2) Branch length: Also known as genetic variation or evolutionary distance. Represents the degree of change in evolutionary branches; shorter lengths indicate smaller differences and closer evolutionary distances. (3) Distance scale: The unit length for measuring differences between organisms or sequences, equivalent to the scale of an evolutionary tree. (4) Self-expansion value: Used to display the reliability of evolutionary tree branches. Typically represented by a number between 0 and 100.

Among the outgroups of the Malvaceae family, *Tilia cordata* and *Tilia miqueliana* from the genus Tilia are relatively closely related to Malvaceae species; whereas *Aquilaria yunnanensis*, *Bixa orellana* are more distantly related. Outgroups such as *Malus pumila*, *Malus prattii*, *Miscanthus sinensis*, and *Rorippa sessiliflora* are the most distantly related to Malvaceae species, with branch self-support rates of 100%, validating the appropriateness of these outgroups (Figure 8).

A maximum-likelihood phylogenetic tree was constructed based on conserved chloroplast CDS sequences from 20 species (Figure 8). *Abelmoschus esculentus* formed a fully supported clade (100% bootstrap) with its congeners *A. sagittifolius* and *A. moschatus*. This Abelmoschus clade grouped with *Hibiscus syriacus* with 100% support, confirming they are sister genera. Within Malvaceae, the Abelmoschus–Hibiscus lineage was successively sister to the clades containing Gossypium (cotton) and Malva, all with high support (≥95%).

## 3. Discussion

### 3.1. Conservation and Uniqueness of Genomic Structure

This study utilized the breeding material ‘Gan Kui No. 1’as the test material and employed Illumina high-throughput sequencing technology to sequence its complete chloroplast genome. Unlike Yan Liu et al., who compared three *Abelmoschus esculentus* varieties, this experiment focused on a single variety and conducted in-depth analyses, including repetitive sequence analysis, codon preference analysis, Ka/Ks analysis, nucleotide diversity analysis, pi analysis, and boundary analysis.

The assembled *Abelmoschus esculentus* chloroplast genome exhibits a typical tetrad structure comprising a large single-copy region (LSC), inverted repeat region (IR), and small single-copy region (SSC), with a total length of approximately 163,121 bp. The length distribution of the LSC region (88,071 bp), IR region (28,009 bp), and SSC region (19,032 bp). This length distribution aligns with previously reported Malvaceae species, such as Hibiscus syriacus (total length 161,025 bp, IR region 25,745 bp) and Gossypium barbadense (total length 160,317 bp, IR region 25,591 bp). This structural framework confirms the long-term evolutionary conservation of chloroplast genome architecture in angiosperms [14,15].

Meanwhile, the chloroplast genome of *Abelmoschus esculentus* encodes a total of 132 genes, comprising 37 tRNA genes, 8 rRNA genes, and 87 mRNA genes, with no pseudogenes detected. Functional classification reveals genes involved in photosynthesis, self-replication, and other functions, exhibiting high homology with closely related species. Among these, the sequence identity of photosystem core genes (psaA, psbB, rbcL) and ribosomal protein genes (rpl2, rps12) exceeded 90%, further demonstrating the evolutionary constraints on core functions within the chloroplast genome as a “semi-autonomous genetic unit.” [16,17].

Systematic comparison across Malvaceae reveals a spectrum of IR sizes: while Abelmoschus species (*A. esculentus*, *A. moschatus*) and *Hibiscus syriacus* possess relatively expanded IRs (28,009 bp and 25,745 bp, respectively), those in Gossypium barbadense and Malva verticillata are more contracted (25,591 bp and 25,107 bp) [18]. This IR expansion in Abelmoschus is not an isolated feature but is linked to specific boundary shifts [19]. As illustrated in Figure 7, genes such as ycf1 and ndhF extend into the IR regions, a pattern convergent in some Hibiscus species but absent in Malva. IR expansion is a recognized evolutionary mechanism that can enhance genomic stability through increased copy number of essential genes (e.g., rRNA operons) and facilitate efficient DNA repair via copy-dependent recombination [20]. Therefore, the shared trait of a relatively large IR may contribute to the genomic robustness of the Abelmoschus–Hibiscus lineage.

Beyond IR dynamics, the distribution of mutation hotspots offers insights into evolutionary rates. Our Pi analysis corroborates that the SSC region is a hypervariable zone, with peaks near *ndhF* and *ycf1*, a pattern consistently observed in *A. moschatus* and other malvaceous species. A more unusual feature is the exceptionally high density of Simple Sequence Repeats (SSRs) in the LSC region of *A. esculentus*. With 236 SSRs (68.6% of the total), the LSC of ‘Gan Kui No. 1’ harbors a significantly richer SSR repertoire than its IR regions (32 each) and appears denser than reports for some Gossypium and Malva species [16,17]. This pronounced asymmetry suggests that the LSC, free from the constraints of copy correction, is a more permissive site for the accumulation of repetitive microsatellites. These polymorphic cpSSRs are not only valuable molecular markers for resolving intraspecific phylogeny and germplasm fingerprinting, but their inherent mutability may also contribute to the generation of regulatory or structural variation upon which selection can act [21,22].

### 3.2. Biological Interpretation of Function-Related Traits

The coding frames of photosynthesis-related genes in the chloroplast genome of *Abelmoschus esculentus*, such as those for Photosystem I/II, NADH dehydrogenase, cytochrome b_6_f, and ATP synthase, are intact. These genes cover the entire photosynthetic pathway, including electron transport, ATP synthesis, and carbon fixation. This sequence consistency reflects strong evolutionary constraints on the photosynthetic function of these genes across Malvaceae species. Purifying selection on core photosynthetic genes ensures the fundamental functional stability of light reactions [23].

Beyond the conservation patterns revealed by codon preference and selective pressure analyses, systematic comparisons with closely related species highlight potential adaptive features in the *Abelmoschus esculentus* chloroplast genome concerning energy metabolism-related genes. We observed that the *atpF* gene, encoding the F0 subunit of ATP synthase, exhibits significant positive selection signals (Ka/Ks > 1) in okra. This contrasts with the predominantly purifying selection observed for this gene in Hibiscus syriacus (Ka/Ks ≈ 0.85) and Gossypium hirsutum (Ka/Ks ≈ 0.79) [24]. This interspecific divergence suggests that atpF may have undergone unique adaptive evolution within the okra lineage. Changes in its amino acid sequence may have optimized proton transport, thereby enhancing photosynthetic phosphorylation capacity under rapid growth or photostress conditions [25]. Further analysis of the rbcL gene encoding the large subunit of ribulose-1,5-bisphosphate carboxylase/oxygenase reveals a notable non-synonymous mutation near the critical carboxylase active site in *Abelmoschus esculentus* (compared to *Abelmoschus moschatus* and *Hibiscus syriacus*), despite overall strong purifying selection. This mutation has been reported in multiple C4 plant *rbcLs* to be associated with enhanced carboxylation efficiency [26]. Although okra is a C3 plant, whether this mutation confers higher carbon assimilation potential—particularly under high-temperature conditions—remains to be functionally validated. These findings link genomic variation to potential improvements in photosynthetic performance, providing molecular clues for understanding the physiological basis of okra as a high-yielding vegetable crop. Among the 19 amino acids encoded by a total of 22,797 codons, the codon preference in the *Abelmoschus esculentus* chloroplast genome exhibits a higher frequency of codons ending with A/U. For instance, alanine (*Ala*) shows a preference for GCU, while lysine (*Lys*) favors AAA. This pattern aligns with the codon usage characteristics observed in most terrestrial plants [27]. From a translational mechanism perspective, the tRNA preference for A/U-terminated codons in chloroplasts should serve as a regulatory mechanism for protein synthesis efficiency [28].

This codon preference is particularly pronounced in photosynthesis-related genes, such as *rbcL*, whose high-frequency codons all terminate with A/U. This suggests that *Abelmoschus esculentus* optimizes codon usage to prioritize efficient expression of core photosynthetic genes. This adaptation is crucial for the growth and development of *Abelmoschus esculentus* as a high-photosynthetic-efficiency crop, reflecting the coevolution of gene expression regulation and physiological demands in functional genes [29].

### 3.3. Distribution Characteristics of Repetitive Sequences

A total of 91 scattered repetitive sequences were identified in the *Abelmoschus esculentus* chloroplast genome, indicating that short repetitive sequences constitute the predominant form of repetitive sequences in the *Abelmoschus esculentus* chloroplast genome. Reverse repeats (R) were the second most common (24, representing 26.4%), while complementary repeats (C) were the least frequent (11, representing 12.1%). The types and distribution of *Abelmoschus esculentus* repetitive sequences exhibit significant preferences, showing marked differences from the repetitive sequence characteristics of Malvaceae genus Gossypium species such as *Gossypium hirsutum* and *Gossypium barbadense*: Scattered repetitive sequences in the chloroplast genomes of Gossypium species are the most common type among the three categories, totaling 1204 sequences, accounting for 42.10% of all repetitive sequences; Palindromic repeats numbered 779, accounting for 27.24%; Tandem repeats totaled 877, representing 30.66% [21]. Differences in this type and abundance may stem from species-specific variations in genomic recombination mechanisms and repair systems.

A total of 344 SSR loci (cpSSR) were detected in the *Abelmoschus esculentus* chloroplast genome, exhibiting significant non-uniform distribution across the genome. Large single-copy regions (LSC) totaled 236 sites, while small single-copy regions (SSC) contained 44 sites. Both inverted repeat sequences, *IRa* and *IRb*, harbored 32 sites each. The abnormal enrichment of SSRs in the LSC region is not random; their distribution spatially overlaps with areas of high nucleotide diversity (Peak Pi values), particularly in intergenic regions such as psbK-psbI and atpF-atpH. These regions typically serve as evolutionary “mutation hotspots.” The high density of A/T-dominated single-nucleotide repeats may significantly elevate local mutation rates through the slipping mismatch mechanism, thereby providing abundant raw material for driving genomic microevolution [22]. Functionally, these variable SSRs located in gene flanking regions may regulate the expression plasticity of adjacent photosynthetic genes (e.g., *atpF*) by influencing promoter activity or mRNA stability, potentially conferring adaptive value in responding to environmental fluctuations [30]. Therefore, the LSC region of okra chloroplasts is typically a hotspot for sequence variation. Its unique SSR distribution pattern may indirectly contribute to the species’ adaptability to diverse growth environments by regulating gene expression, offering a novel perspective for understanding chloroplast evolution through the lens of epigenomic dynamics.

### 3.4. Gene Dialogue and Evolutionary Dynamics Among Organelles

Plant organelle genomes (chloroplasts and mitochondria) do not evolve in isolation; active sequence migration and intracellular gene transfer (IGT) occur between them [31]. Recent studies of the okra mitochondrial genome reveal the presence of numerous mitochondrial-derived chloroplast transfer fragments (MTPTs), including complete or nearly complete chloroplast genes such as *psaA*, *rps7*, and *psbJ*. Li et al. (2022) reported the phenomenon of a gene cluster composed of *psbJ*, *psbL*, *psbF*, *psbE* [32]. This finding may hold intriguing evolutionary connections to the chloroplast genomic structural features observed in the present study. The gene cluster has undergone pseudogenization within the mitochondria, suggesting potential loss or alteration of function. This specific instance of intergenic transfer (IGT) provides a microscopic case study for understanding the coevolution of organelle genomes in okra. This study reveals a relatively expanded IR region (28,009 bp) in the chloroplast genome of ‘Gan Kui No. 1’. We hypothesize that the expansion of the IR region may have increased genomic instability or created hotspots for homologous recombination, while the high density of SSRs in the LSC region may have functioned as a “sequence module.” Together, these factors may have historically facilitated the migration of specific sequence fragments to mitochondria. This inter-organelle gene flow may represent a deep genomic evolutionary strategy enabling *Abelmoschus esculentus*’s adaptation to its extensive geographic distribution.

### 3.5. The Taxonomic Significance of Phylogenetic Results

Based on shared CDS sequences, the phylogenetic tree constructed in this study shows that *Abelmoschus esculentus* clusters together with its congeneric species *Abelmoschus sagittifolius* and *Abelmoschus moschatus* in a branch with 100% bootstrap support. This result indicates extremely high genetic consistency and close phylogenetic relationships among species within the genus at the chloroplast genome level. Numerous previous studies have also fully substantiated this conclusion [11]. The 20 species selected for this study encompass major groups within the Malvaceae family as well as extramalvaceous taxa. The phylogenetic tree constructed based on shared CDS sequences exhibits a majority branch support rate of 100%, significantly exceeding the standard of ≥70% support rate commonly used in taxonomic research [33], indicating that the results possess an extremely high degree of credibility.

The genus Hibiscus and the genus Abelmoschus form a sister clade with identical support of 100%, confirming them as the most closely related groups within the Malvaceae family. This finding also corrects earlier research suggesting that the genus Malabar-chickpea was more closely related to the genus Gossypium [34]. Within the Malvaceae family, the AbelmoschusHibiscus clade clusters sequentially with Gossypium (cotton) and Malva, with a support rate ≥ 95%, reflecting an evolutionary pattern centered on the Abelmoschus–Hibiscus core group within the family. At the cross-family level, Tilia and Firmiana show relatively close relationships with Malvaceae species, while outgroups such as Malus and Miscanthus are distantly related to the Malvaceae clade. Notably, all outgroup branches exhibit 100% support rates, validating the reliability of the phylogenetic tree topology.

## 4. Materials and Methods

### 4.1. Materials and Sequencing

Fresh leaves of the okra cultivar Abelmoschus esculentus ‘Gan Kui No. 1’ were collected, immediately frozen in liquid nitrogen, and stored at −80 °C. Total genomic DNA was extracted using a plant DNA extraction kit (Jisihuiyuan D312, Nanjing Jisihuiyuan Biotechnology Co., Ltd., Nanjing, China). Paired-end (2 × 150 bp) sequencing was performed on an Illumina NovaSeq X Plus platform.

### 4.2. Chloroplast Genome Assembly and Annotation

Raw sequencing reads were quality-controlled using fastp (v0.20.0) [35]. Adaptors and low-quality bases (Q < 20) were trimmed, and reads containing more than 5% ambiguous nucleotides (N) were discarded to generate clean data for downstream assembly. MAFFT (v3.10.1) [36] software was employed for de novo assembly independent of reference genomes, with kmer parameters set to 55, 87, and 121. Due to the limitations of second-generation sequencing technology, the assembly workflow was designed as a multi-step iterative process to obtain complete circular sequences. The chloroplast genome assembly first utilizes SPAdes (v3.10.1) software to assemble the cpDNA sequence, yielding the SEED sequence of the genome; Subsequently, the SEED sequence underwent iterative kmer extension. If the extension yielded only a single contig, it was designated as the preliminary genome and proceeded directly to the subsequent correction phase. If multiple contigs were generated, SSPACE v2.0 software [37] was first used to assemble these contigs into scaffolds. Subsequently, GapFiller v2.1.1 software [38] was employed to fill gaps within the scaffolds. If gaps persist after filling, primers are designed for PCR sequencing, and reassembly is repeated until a gap-free preliminary circular sequence is obtained. After obtaining the preliminary genome (including directly acquired single contigs and gap-free circular sequences), sequencing reads are mapped back to this sequence for genome correction. Finally, the corrected sequence undergoes coordinate realignment based on the standard chloroplast structure, yielding the complete circular chloroplast genome sequence. Alternatively, specialized chloroplast assembly software like GetOrganelle can be employed to further enhance assembly efficiency.

To ensure the accuracy of chloroplast assembly results. First, clean reads are mapped back to the assembled genome sequence. Core metrics such as genome coverage and insert size are statistically analyzed to validate the completeness and reliability of the assembled sequence coverage. Next, the assembled genome is aligned with the reference genome of a closely related species to assess the distribution of conserved genomic regions and identify potential rearrangements. Finally, the analysis focuses on structural details, particularly comparing differences between the assembled and reference genomes in key structures such as inverted repeat regions (IR regions), to comprehensively confirm the accuracy of the assembly results.

Gene annotation was performed using a dual approach: protein-coding genes were predicted with Prodigal (v2.6.3), while tRNAs and rRNAs were identified with Aragorn (v1.2.38) and HMMer (v3.1b2), respectively [39,40,41]. Alternatively, a second annotation result can be obtained by performing homology alignment using BLAST (v2.6) based on gene sequences from closely related species in NCBI [42]. Discrepancies were manually curated to define precise gene boundaries. The annotated genome map was drawn using OGDRAW (v1.3.1) [43].

### 4.3. Codon Usage and Repeat Sequence Analysis

RSCU values were calculated for all protein-coding genes using a custom Perl script.

Repetitive sequences were identified using the vmatch (v2.3.0) [38] software in conjunction with a Perl script. Parameters were set as follows: minimum length of 30 bp, Hamming distance of 3, and identification of four types: forward, palindromic, reverse, and complementary [44].

For cpSSR analysis using MISA, the parameters are set as follows: single base ≥ 8, double base ≥ 5, triple to sextuple base ≥ 3.

### 4.4. Selective Pressure and Nucleotide Diversity Analysis

To evaluate selective pressures, we aligned the protein-coding sequences of *A. esculentus* with their orthologs from two closely related species (*Abelmoschus moschatus* and *Hibiscus syriacus*). Multiple sequence alignments were generated with MAFFT (v3.10.1) [45], and the non-synonymous (Ka) to synonymous (Ks) substitution ratios were computed using KaKs_Calculator (v2.0) [46] under the MLWL model for plant plastid codes. To assess sequence variation, we aligned the complete chloroplast genomes of *A. esculentus*, *A. moschatus*, *A. sagittifolius*, and *H. syriacus* using MAFFT. Nucleotide diversity (Pi) for each gene region was calculated using DnaSP (v5) [47] with a sliding window of 200 bp and a step size of 50 bp.

### 4.5. IR Boundary and Comparative Genomics Analysis

The exact boundaries between the inverted repeat (IR) regions and the large/small single-copy (LSC/SSC) regions were determined by manual inspection of the annotated genome. A comparative visualization of these boundaries across five Malvaceae species (*A. esculentus*, *A. moschatus*, *H. syriacus*, *Gossypium hirsutum*, and *Malva verticillata*) was generated using a Perl script with the SVG module to illustrate IR expansion/contraction events. Whole-chloroplast genome comparisons and visualization of structural rearrangements were performed using CGView [48]. Synteny and large-scale structural conservation were assessed with Mauve (v2.3.1) [49] under default parameters to detect potential inversions or rearrangements among the analyzed species.

### 4.6. Methods for Constructing Phylogenetic Trees and Parameter Settings

Phylogenetic trees were constructed based on whole-genome sequences. The chloroplast circular genomes of each species were aligned using MAFFT (v3.10.1) with a unified start position, and unreliable regions were trimmed using trimAl (v1.4.rev15) [50]. Subsequently, a maximum likelihood phylogenetic tree was constructed using RAxML (v8.2.10) software with the GTRGAMMA model and 1000 rapid bootstrap tests [50]. Including three species of the genus Abelmoschus: *Abelmoschus esculentus*, *Abelmoschus moschatus*, and *Abelmoschus sagittifolius*; *Malva cathayensis* and *Malva verticillata* of the genus Malva. Less closely related species include Corchorus capsularis; *Firmiana simplex* and *Firmiana major* from the genus Firmiana; *Theobroma cacao* from the genus Theobroma; *Hibiscus syriacus* from the genus Hibiscus; *Tilia cordata* and *Tilia miqueliana* from the genus Tilia; and *Gossypium hirsutum* and *Gossypium barbadense* from the genus Gossypium. The most distantly related species include *Miscanthus sinensis*, *Rorippa sessiliflora*, *Bixa orellana*, *Aquilaria yunnanensis*, *Malus pumila*, and *Malus prattii*.

## 5. Conclusions

This study utilized the Abelmoschus esculentus cultivar Gan Kui No. 1 as the mate-rial, employing Illumina high-throughput sequencing technology to complete the sequencing, assembly, and annotation of its complete chloroplast genome. We systematically conducted genomic characterization and phylogenetic analysis.

The full-length genome of Abelmoschus esculentus chloroplasts spans 163,121 bp. Repeat sequences predominantly consist of short repeats ranging from 30 to 37 bp. The 344 SSR loci are unevenly distributed across the genome, with the LSC region accounting for 65.4% of the total. Ka/Ks analysis indicates that genes such as atpF undergo positive selection, contributing to environmental adaptation. Analysis reveals that the LSC and SSC regions are active areas of genetic variation, while the IR region exhibits extremely low variation due to structural conservation. Identification of positively selected genes (e.g., atpF) suggests potential adaptations in energy metabolism; the preference for A/U stop codons (particularly in core photosynthetic genes) may reflect optimization of translation efficiency; comparative analysis of intergenic regions (IRs) reveals dynamic expansion/contraction events within the Malvaceae family, where the expanded IR regions in Malva may contribute to genomic stability. Furthermore, the clustering of SSRs within hypervariable regions of LSC and SSC domains highlights these regions as mutation hotspots, potentially serving as sources of genetic diversity and regulatory variation.

Phylogenetic analysis robustly places *A. esculentus* within a fully supported clade containing the closely related species *A. sagittifolius* and *A. moschatus*, confirming its sister group relationship with the genus Hibiscus. This provides definitive genomic evidence for the classification of the kapok-hibiscus lineage within Malvaceae.

In summary, this study elucidates the structural characteristics, sequence variation patterns, and phylogenetic position of the *Abelmoschus esculentus* chloroplast genome. It provides a crucial theoretical foundation for *Abelmoschus esculentus* germplasm resource exploration, variety improvement, and genetic breeding, while also offering key reference data for evolutionary mechanisms and taxonomic research in Malvaceae plants.

## Figures and Tables

**Table 1:** Base composition characteristics of different sequence regions (LSC, SSC, IRa, IRb).

Region	A Content/%	C Content/%	G Content/%	T Content/%	GC Content/%	Base Length/bp
LSC	32.08	17.78	16.77	33.37	34.55	88,071
SSC	33.87	16.36	15.12	34.64	31.48	19,032
IRa	29.53	21.66	20.30	28.51	41.97	28,009
IRb	28.51	20.30	21.66	29.53	41.97	28,009
Total volume	31.24	18.71	18.02	32.02	36.74	163,121

**Table 2:** Gene annotation of the chloroplast genome of *Abelmoschus esculentus* L.

Category	Gene Group	Gene Name
Photosynthesis	Subunits of photosystem I	*psaA*, *psaB*, *psaC*, *psaI*, *psaJ*
	Subunits of photosystem II	*psbA*, *psbB*, *psbC*, *psbD*, *psbE*, *psbF*, *psbH*, *psbI*, *psbJ*, *psbK*, *psbL*, *psbM*, *psbN*, *psbT*, *psbZ*
	Subunits of NADH dehydrogenase	ndhA*, ndhB*(2), *ndhC*, *ndhD*, *ndhE*, *ndhF*, *ndhG*, *ndhH*, *ndhI*, *ndhJ*, *ndhK*
	Subunits of cytochrome b/f complex	*petA*, petB*, petD*, *petG*, *petL*, *petN*
	Subunits of ATP synthase	*atpA*, *atpB*, *atpE*, atpF*, *atpH*, *atpI*
	Large subunit of rubisco	*rbcL*
	Subunits photochlorophyllide reductase	-
Self-replication	Proteins of the large ribosomal subunit	*rpl14*, rpl16*, rpl2*(2), *rpl20*, *rpl22(2)*, *rpl23(2)*, *rpl32*, *rpl33*, *rpl36*
	Proteins of the small ribosomal subunit	*rps11*, rps12**(2), *rps14*, *rps15*, rps16*, *rps18*, *rps19(2)*, *rps2*, *rps3(2)*, *rps4*, *rps7(2)*, *rps8*
	Subunits of RNA polymerase	*rpoA*, *rpoB*, rpoC1*, *rpoC2*
	Ribosomal RNAs	*rrn16(2)*, *rrn23(2)*, *rrn4.5(2)*, *rrn5(2)*
	Transfer RNAs	trnA-UGC*(2), *trnC-GCA*, *trnD-GUC*, *trnE-UUC*, *trnF-GAA*, *trnG-GCC*, trnG-UCC*, *trnH-GUG*, *trnI-CAU(2)*, trnI-GAU*(2), trnK-UUU*, *trnL-CAA(2)*, trnL-UAA*, *trnL-UAG*, *trnM-CAU*, *trnN-GUU(2)*, *trnP-UGG*, *trnQ-UUG*, *trnR-ACG(2)*, *trnR-UCU*, *trnS-GCU*, *trnS-GGA*, *trnS-UGA*, *trnT-GGU*, *trnT-UGU*, *trnV-GAC(2)*, trnV-UAC*, *trnW-CCA*, *trnY-GUA*, *trnfM-CAU*
Other genes	Maturase	*matK*
	Protease	clpP**
	Envelope membrane protein	*cemA*
	Acetyl-CoA carboxylase	*accD*
	c-type cytochrome synthesis gene	*ccsA*
	Translation initiation factor	-
	other	-
Genes of unknown function	Conserved hypothetical chloroplast ORF	*ycf1*, *ycf2(2)*, ycf3**, *ycf4*

Note: Gene*: Contains one intron; Gene**: Contains two introns; Gene(2): Gene with copy number greater than 1, with copy number indicated in parentheses.

**Table 3:** Relative synonymous codon usage analysis of *Abelmoschus esculentus* L.

Symbol	Codon	No.	RSCU	Symbol	Codon	No.	RSCU	Symbol	Codon	No.	RSCU
Ter	UAA	43	1.6539	Lys	AAA	897	1.5088	Arg	AGA	399	1.7616
Ter	UAG	17	0.6537	Lys	AAG	292	0.4912	Arg	AGG	148	0.6534
Ter	UGA	18	0.6924	Leu	CUA	332	0.8286	Arg	CGA	317	1.3998
Ala	GCA	332	1.0312	Leu	CUC	148	0.3696	Arg	CGC	111	0.4902
Ala	GCC	213	0.6616	Leu	CUG	152	0.3792	Arg	CGG	89	0.393
Ala	GCG	169	0.5248	Leu	CUU	492	1.2282	Arg	CGU	295	1.3026
Ala	GCU	574	1.7828	Leu	UUA	789	1.9692	Ser	AGC	103	0.3654
Cys	UGC	63	0.496	Leu	UUG	491	1.2252	Ser	AGU	343	1.2168
Cys	UGU	191	1.504	Met	AUA	0	0	Ser	UCA	356	1.263
Asp	GAC	185	0.4062	Met	AUC	0	0	Ser	UCC	259	0.9192
Asp	GAU	726	1.5938	Met	AUG	526	7	Ser	UCG	147	0.5214
Glu	GAA	898	1.508	Met	AUU	0	0	Ser	UCU	483	1.7136
Glu	GAG	293	0.492	Met	CUG	0	0	Thr	ACA	353	1.2108
Phe	UUC	424	0.6578	Met	GUG	0	0	Thr	ACC	227	0.7788
Phe	UUU	865	1.3422	Met	UUG	0	0	Thr	ACG	138	0.4736
Gly	GGA	619	1.5368	Asn	AAC	250	0.4638	Thr	ACU	448	1.5368
Gly	GGC	173	0.4296	Asn	AAU	828	1.5362	Val	GUA	470	1.5088
Gly	GGG	290	0.72	Pro	CCA	258	1.1048	Val	GUC	151	0.4848
Gly	GGU	529	1.3136	Pro	CCC	174	0.7452	Val	GUG	170	0.5456
His	CAC	139	0.5054	Pro	CCG	131	0.5612	Val	GUU	455	1.4608
His	CAU	411	1.4946	Pro	CCU	371	1.5888	Trp	UGG	403	1
Ile	AUA	613	0.9354	Gln	CAA	625	1.549	Tyr	UAC	172	0.4018
Ile	AUC	371	0.5661	Gln	CAG	182	0.451	Tyr	UAU	684	1.5982
Ile	AUU	982	1.4985								

Note: Symbol: Three-letter amino acid abbreviation, denotes stop codon; Codon: Codon; No.: Number of codons; RSCU: Codon preference.

**Table 4:** Analysis of scattered sequence repeats in the chloroplast genome of *Abelmoschus esculentus* L.

Length	F	P	R	C	Total
30	8	5	6	7	26
31	0	5	4	0	9
32	2	0	8	2	12
33	2	3	1	0	6
34	3	2	4	1	10
35	1	1	0	1	3
36	4	3	0	0	7
37	1	2	0	0	3
39	1	0	1	0	2
40	0	1	0	0	1
41	2	0	0	0	2
43	1	2	0	0	3
46	0	1	0	0	1
52	1	0	0	0	1
53	2	2	0	0	4
28,009	0	1	0	0	1
Total	28	28	24	11	91

Note: Length represents the length of the repetitive sequence; F denotes forward repeats, P denotes palindromic repeats, R denotes reverse repeats, and C denotes complementary repeats; Total represents the number of all repeats.

## Data Availability

The original contributions presented in this study are included in the article. Further inquiries can be directed to the corresponding authors. All original data (including sequencing reads and annotated genomes) supporting the reported results have been submitted to NCBI GenBank (Submission ID: PX590535).
